# Supplementary material for: Solvent-driven fractional crystallization for atom-efficient separation of metal salts from permanent magnet leachates
Source: Nat Commun. 2022 Jul 1;13:3789. doi: 10.1038/s41467-022-31499-7 (PMC9249736; doi:10.1038/s41467-022-31499-7)
Supplement: Supplementary file 1 — Supplementary Information [file 41467_2022_31499_MOESM1_ESM.pdf]

Supplementary Information for:

Solvent-Driven Fractional Crystallization for Atom-Efficient Separation of Metal Salts from Permanent Magnet Leachates

Caleb Stetson,[a] Denis Prodius,[b] Hyeonseok Lee,[a] Christopher Orme,[a] Byron White,[a] Harry Rollins,[a] Daniel Ginosar,[a] Ikenna C. Nlebedim,[b] Aaron D. Wilson[a,*]

[a] *Critical Materials Institute, Idaho National Laboratory, 1955 N Fremont Ave, Idaho Falls, ID, USA 83415*
[b] *Critical Materials Institute, Ames Laboratory, US Department of Energy, Ames, IA50011-3020, USA*
*Corresponding Author, Aaron.Wilson@inl.gov

Keywords: hydrometallurgy, crystallization, dimethyl ether, rare earth elements, atom economy, green chemistry

Supplementary Note 1: Example Experiment

100 mL of a mixed metal sulfate leachate with initial metal concentrations of 42.857 g/L Co, 20.128 g/L Sm, and 3.215 g/L Fe was introduced to the reaction chamber via the gear pump; water was recirculated in the outer chamber at 20° C. DME gas was introduced to the reaction chamber and purged 5 times to remove partial pressures of atmospheric gas. After purging, chamber was pressurized with DME gas from the gas tank to 62 psi above atmosphere, with the gear pump recirculating gas from the chamber headspace through the aqueous solution via a 1/8” Teflon tube. As DME dissolves into the system, volume of the aqueous solution expands (~25%), and the solution becomes visibly turbid after ~15 minutes of gas recirculation. Several minutes after the appearance of turbidity, visible crystal growth begins on the nucleation scaffold. As crystal growth begins, a partitioning of the aqueous solution into an enriched phase at the bottom of the reaction chamber, and a depleted phase above the enriched phase is induced by chamber geometry and gas sparging.

Aqueous samples were obtained via flow control at Swagelok two-way ball valves at the bottom of the reaction chamber during the experiment. Upon removal of residual aqueous solution and chamber depressurization, the apparatus was disassembled, and the stainless-steel nucleation scaffold was extracted. Crystals were then mechanically removed from the scaffold for further characterization. Aqueous samples were analyzed with ultraviolet-visible (UV-Vis) spectroscopy then transferred for inductively coupled plasma optical emission spectroscopy (ICP-OES) to measure Co, Sm, and Fe concentration. Solid samples were investigated with powder X-ray diffraction (XRD) to determine crystal structure and digested with HCl for ICP-OES analysis of Co, Sm, and Fe.

Supplementary Table 1. ICP-OES Metal Concentrations for Original Leachates, Treated Solutions and Digested Solid Samples, Listed in Order of Appearance

Figure	Sample	ICP-OES Concentration (mg/L), *Metal Mass Percent*[1]					
		Co	Fe	Sm	Nd	Pr	Dy
2b	Sm-Co Original Leachate	42857<br>*64.74%*	3215<br>*4.86%*	20128<br>*30.40%*			
2c	Nd-Fe-B Original Leachate	7704<br>*13.15%*	22765<br>*38.85%*	3050<br>*5.20%*	17437<br>*29.75%*	5758<br>*9.83%*	1886<br>*3.22%*
3a	Co-Rich Solids, 20°C Treatment	195580<br>*91.32%*	17631<br>*8.23%*	961<br>*0.45%*			
3a	Sm-Rich Solids, 31°C Treatment	10<br>*0.29%*	10<br>*0.28%*	3466<br>*99.43%*			
3b	Ln-Rich Solids, 31°C Treatment	10<br>*0.41%*	43<br>*1.77%*	467<br>*19.23%*	1393<br>*57.34%*	446<br>*18.38%*	69<br>*2.86%*
4b	Sm-Co Original Leachate	42857<br>*64.74%*	3215<br>*4.86%*	20128<br>*30.40%*			
4b	Sm-Co Stage 1 Solids	10121<br>*77.01%*	624<br>*4.75%*	2398<br>*18.24%*			
4b	Sm-Co Stage 2 Solids	16352<br>*93.22%*	1114<br>*6.35%*	75<br>*0.43%*			
4c	Nd-Fe-B Original Leachate	7704<br>*13.15%*	22765<br>*38.85%*	3050<br>*5.20%*	17437<br>*29.75%*	5758<br>*9.83%*	1886<br>*3.22%*
4c	Nd-Fe-B Stage 1 Solids	3836<br>*22.42%*	11781<br>*68.88%*	310<br>*1.81%*	825<br>*4.82%*	274<br>*1.60%*	78<br>*0.46%*
4c	Nd-Fe-B Stage 2 Solids	1439<br>*24.16%*	4509<br>*75.67%*	3<br>*0.04%*	5<br>*0.09%*	1<br>*0.02%*	1<br>*0.02%*
S2a	Sm-Co Original Leachate	42857<br>*64.74%*	3215<br>*4.86%*	20128<br>*30.40%*			
S2a	Sm-Co Leachate Treated 20°C	1773<br>*76.53%*	111<br>*4.78%*	433<br>*18.69%*			
S2a	Sm-Co Leachate Treated 31°C	35066<br>*77.25%*	2784<br>*6.13%*	7541<br>*16.61%*			
S2b	Nd-Fe-B Original Leachate	7704<br>*13.15%*	22765<br>*38.85%*	3050<br>*5.20%*	17437<br>*29.75%*	5758<br>*9.83%*	1886<br>*3.22%*
S2b	Nd-Fe-B Leachate Treated 31°C	4703<br>*20.45%*	13510<br>*58.73%*	908<br>*3.95%*	2572<br>*11.18%*	801<br>*3.48%*	511<br>*2.22%*
S4	Sm-Co Original Leachate	42857<br>*64.74%*	3215<br>*4.86%*	20128<br>*30.40%*			
S4	Sm-Co Leachate 31°C Pass	45238<br>*79.24%*	3300<br>*5.78%*	8555<br>*14.98%*			
S4	Sm-Co Leachate 20°C Pass	1773<br>*76.53%*	111<br>*4.78%*	433<br>*18.98%*			

[1] Metal mass percentages calculated from ICP-OES concentrations are given beneath concentration data in italics.

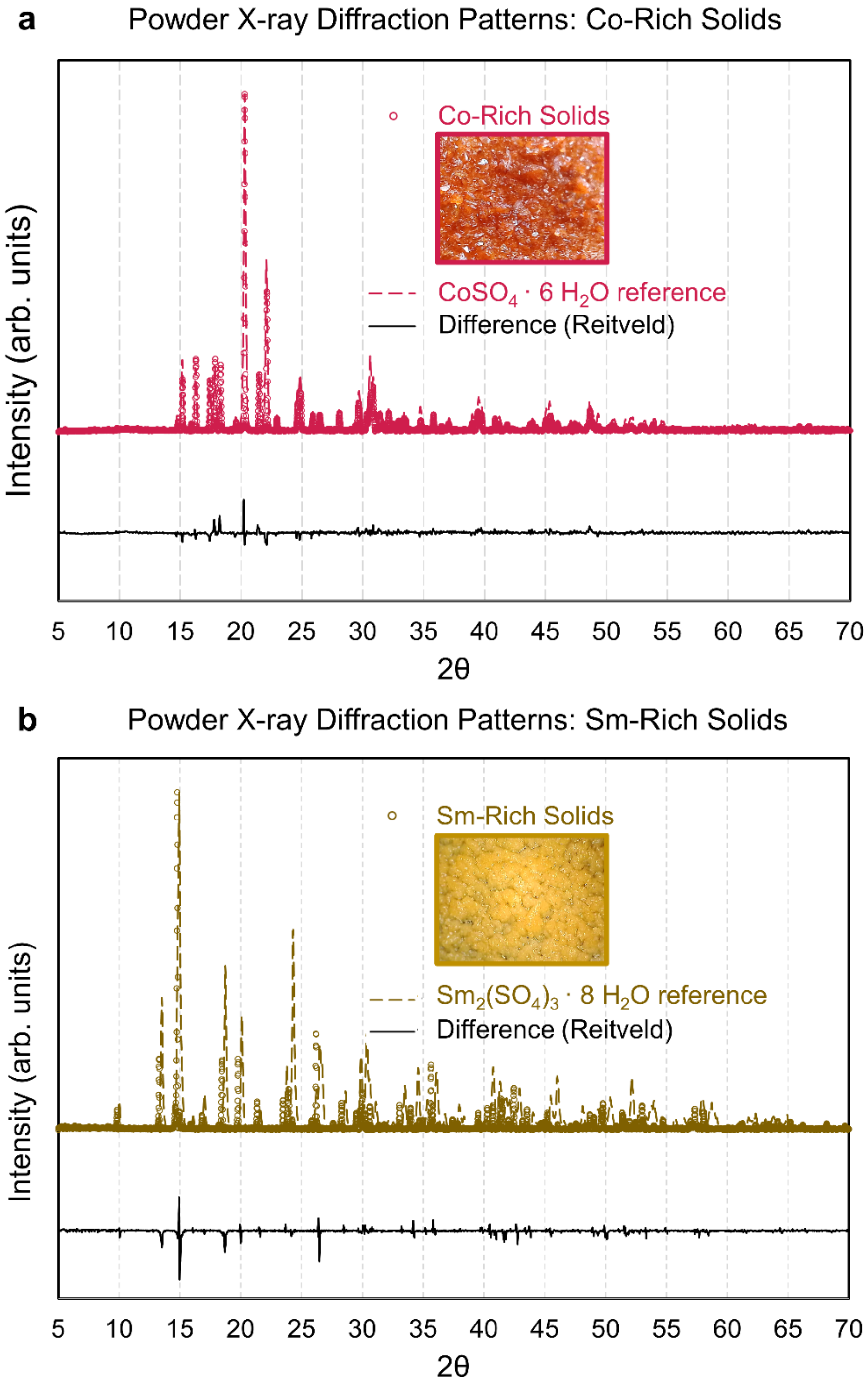

Supplementary Figure 1. Structural identification of DME-FC solid products. a Powder X-ray diffraction (XRD) patterns for Co-rich solids and **b** Sm-rich solids. Known powder references[1,2] are given as traces; difference between the observed and calculated XRD patterns obtained by Reitveld refinement are given below diffraction data. Similarity of XRD patterns from solid products to known sulfate references indicates that the solid products are precipitated as sulfates, chemically equivalent to the solvated metal sulfates in solution.

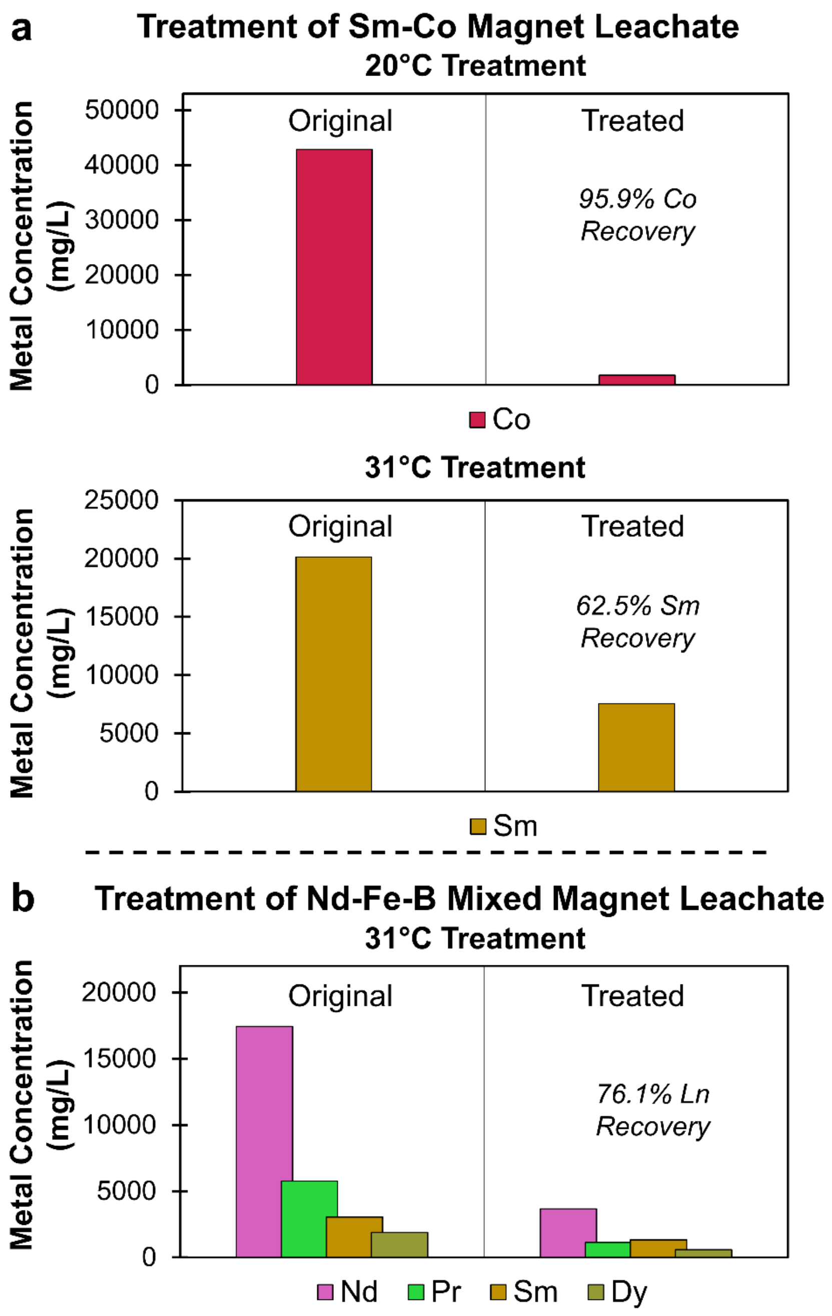

Supplementary Figure 2. Analysis of DME-FC recovery fractions. Comparison of metal concentrations in the original leachates in comparison to treated solutions for the **a** Sm-Co magnet leachate and **b** Nd-Fe-B mixed magnet leachate.

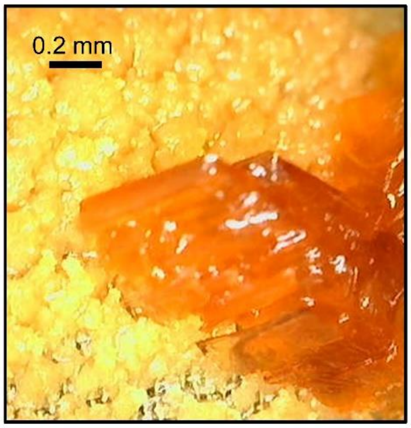

Supplementary Figure 3. Evidence of co-crystallization in DME-FC. Optical image of two classes of precipitates on stainless-steel scaffold after prolonged DME-FC treatment at 23°C. Initially, Co-rich solids were crystallized from solution. After crystallizing the majority of the transition metal fraction (which yields an Sm-rich treated solution), several hours of prolonged exposure to DME resulted Sm-rich solids crystallizing from the solution in spatially distinct locations on the nucleation scaffold. This result is indicative of the complexity that develops in the FC system if DME exposure is extended to longer periods after the initial salt fraction has been crystallized. Moreover, the spatially distinct formation of dissimilar metal salt crystals lends to the hypothesis that solid product crystal structure is important to DME-FC separation factors.

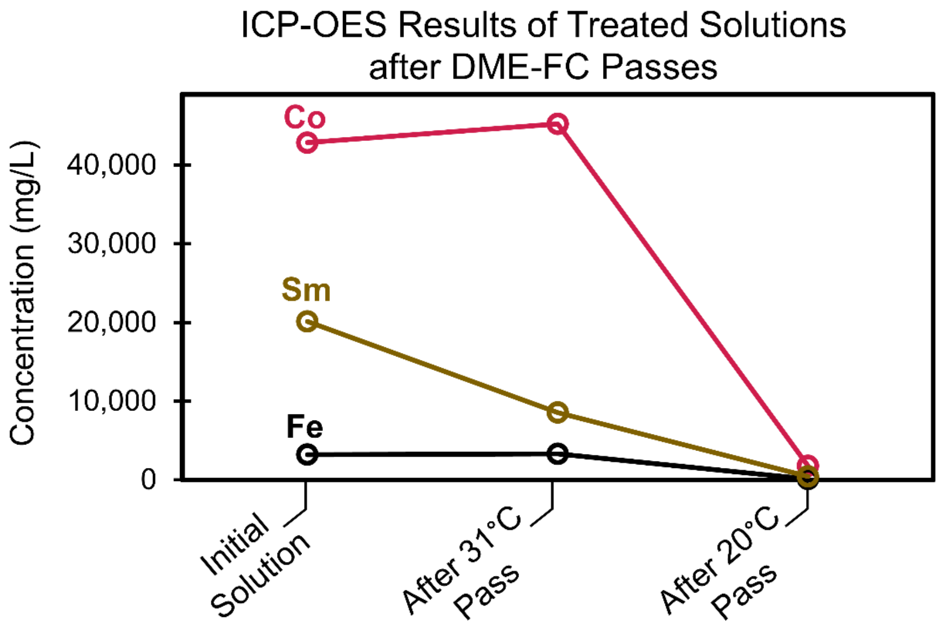

Supplementary Figure 4. Analysis of solutions treated sequentially in DME-FC passes. ICP-OES measurement of initial Sm-Co magnet leachate and treated solutions after successive FC passes from the same solution, initially to precipitate Sm-rich solids at 31°C, followed by treatment at 20°C to precipitate transition metal-rich solids.

References

1 Mobin, S. M. & Mohammad, A. Retention of single crystals of two Co(ii) complexes during chemical reactions and rearrangement. *Dalton transactions* **43**, 13032 (2014).

2 Podberezskaya, N. V. & Borisov, S. V. Refinement of the crystal structure of $Sm_2(SO_4)_3$-$8H_2O$. *Journal of Structural Chemistry* **17**, 164-165 (1976).
